# Identifying influential spreaders in complex networks for disease spread and control

**DOI:** 10.1038/s41598-022-09341-3

**Published:** 2022-04-01

**Authors:** Xiang Wei, Junchan Zhao, Shuai Liu, Yisi Wang

**Affiliations:** 1 Department of Engineering, Honghe University, Honghe, 661100 People’s Republic of China; 2 School of Science, Hunan University of Technology and Business, Changsha, 410205 People’s Republic of China; 3 School of Big Data Science and Application, Chongqing Wenli University, Chongqing, 402160 People’s Republic of China

**Keywords:** Statistical physics, thermodynamics and nonlinear dynamics, Computational science

## Abstract

Identifying influential spreaders is an important task in controlling the spread of information and epidemic diseases in complex networks. Many recent studies have indicated that the identification of influential spreaders is dependent on the spreading dynamics. Finding a general optimal order of node importance ranking is difficult because of the complexity of network structures and the physical background of dynamics. In this paper, we use four metrics, namely, betweenness, degree, H-index, and coreness, to measure the central attributes of nodes for constructing the disease spreading models and target immunization strategies. Numerical simulations show that spreading processes based on betweenness centrality lead to the widest range of propagation and the smallest epidemic threshold for all six networks (including four real networks and two BA scale-free networks generated according to Barabasi–Albert algorithm). The target immunization strategy based on the betweenness centrality of nodes is the most effective for BA scale-free networks but displays poor immune effect for real networks in identifying the most important spreaders for disease control. The immunization strategy based on node degrees is the most effective for the four real networks. Findings show that the target immune strategy based on the betweenness centrality of nodes works best for standard scale-free networks, whereas that based on node degrees works best for other nonstandard scale-free networks. The results can provide insights into understanding the different metrics of measuring node importance in disease transmission and control.

## Introduction

An interesting challenge in network science is understanding the relationship between the system structure and its dynamics. This condition makes it important to find the decisive network structure factors that will allow better control of the system’s functionality^1–4^. Epidemic spreading in complex networks has attracted much attention for a long time, the SIS model for the dynamics of malaria in human populations is studied^5^, it is found that whenever the number of reproduction is greater than 1, the time of disease extinction is exponentially distributed, so it becomes very large in a large population. Mar$\acute{i}$a et al. analyzed the disease dynamics in highly clustered networks with short average path length^6^, they revealed that there was an exponent $p\ne 1$ for which $<S/N>p=R_0^{-1}$. Mayra et al.^7^ constructed a metapopulation model through the network to study the population migration in the case of dengue. The model can determine the flow pattern of the disease in the large region of southeast Mexico. Li et al.^8^ developed the Colombian ZIKV transmission model on the complex network. The model considers the sexual communication and media communication between people in the transmission process, and displays the intrusion area of ZIKV in these two parameter spaces . Many research results are concentrated on determining which network topological attributes are closely related to information and virus transmission^9–11^. In particular, many results focus on the centrality of a topological feature, including betweenness, degree, H-index, and core centralities. These agents are expected to be the most influential expanders because most central nodes can spread their effect to the entire network faster than others. Recently, Kitsak et al.^12^ used different locations of the spreading origin and found evidence to support this hypothesis in the case of an epidemic outbreak. The most influential spreader can be predicted through K-core decomposition analysis. These agents are located at the core of the network and do not need to be closely connected. Silva et al.^13^ studied the correlation between the heterogeneous propagation and central properties of nodes that originally transmitted disease and found that a strong correlation exists between node degree and propagation accessibility. For different dynamic processes, such as the standard rumor models, identifying the most influential spreaders with the same metrics^14^ is impossible. Nine centrality metrics correlating with the rumor and disease spreading capabilities of the nodes were investigated^15^, and the generalized reachability is more related to the propagation process in spatial networks than that in nonspatial networks.

Many studies of disease transmission in complex networks mainly use mean-field method^16,17^, which is simple and easy to understand. The mean-field method is widely used in the analysis and prediction of epidemiology propagation dynamics^18–20^, voting model^21^, and synchronization phenomenon^22,23^. The mean-field method is a method of collectively treating the effects at the level of classes. Assume that the nodes of the same degree behave equally. For homogeneous^24^ and uniform networks (degree distribution obeys Normal or Poisson distribution), scholars applied the homogeneous mean-field method to model analysis. For heterogeneous Barab$\acute{a}$si-Albert (BA) networks^25^, such as scale-free networks, scholars used heterogeneous mean-field methods by assuming that all nodes with equal degree *k* follow the same dynamic process. Many mean-field methods are used to model the spreading process on the basis of the node degree distribution, and the attributes used to characterize the importance of nodes include the metrics of node degree and the metrics of betweenness, H-index, coreness^26,27^. The relationship between degree, H-index, and coreness was displayed in^26^, where degree, H-index, and coreness are the initial, intermediate, and steady states of these sequences, respectively. Since the mean-field method used to describe propagation dynamics assumes that nodes with the same degree have the same dynamics, can the mean-field method be constructed and assume the nodes with the same H-index or coreness or betweenness have the same dynamics?

Although many studies have provided evidence for the presence of influential spreaders in disease transmission, the conclusions are not universal. No consensus is reached under the definition of network centrality because different metrics are used to quantify the centrality of nodes for specific physical background^28^. Controlling the spread of infectious diseases through complex networks, such as the spread of rumors in social interaction and the spread of infection in the population, has attracted increasing attention. Sk et al. proposed a novel mathematical model to forecast COVID-19 and assess control strategies, they found that investing on the quarantined was more effective than isolated people in reducing cases^29^. Li et al. developed a SEIQR difference-equation model to investigate the dynamics of COVID-19. The advantage of this model is that it does not need to estimate the initial value of the model^30^. From the statistical and mathematical analysis of of COVID-19 case reports, human mobility and temporal and spatial changes of transmission control measures, they concluded that China’s control measures made it possible to avoid hundreds of thousands of cases^31^. Considering the impact of blockade and medical resources on the spread of COVID-19 in Wuhan, Sun et al. put forward a dynamic model of epidemic transmission and found that the more abundant medical resources, the smaller the final scale^32^. Immunizing a small number of the highest degree nodes can effectively eliminate virus spreading in scale-free networks^33^. An improved mean-field model to study immunization was proposed by utilizing the degree centrality before and after the immunization^34^, showing that the epidemic threshold for infectious diseases is low. A new measure of centrality that utilizes the coreness and eigenvector centralities was proposed to identify the influential spreaders^35^. The results displayed that this approach is more influential than other benchmarks. Many metrics of node centrality are used to select the nodes for target immunization.

In this study, heterogeneous mean-field propagation models based on node betweenness centrality, degree, H-index, and coreness are constructed. The numerical simulations show that the spreading processes based on betweenness centrality lead to the widest range of propagation processes. With the target immunity for disease transmission, the numerical simulations of uniform and target immunizations based on node betweenness centrality, degree, H-index, and coreness display that the immunization strategy based on betweenness centrality performs competitively for BA scale-free networks, and the immunization strategy based on degree centrality performs competitively in comparison with other strategies for real networks.

## Methods

### Epidemic model construction strategies

The classical susceptible-infected-susceptible (SIS) model^16^ is used to describe the spreading dynamics on heterogeneous network at the level of classes of nodes. Assume that all nodes of the same degree (betweenness, H-index, and *k*-coreness) behave equally. Define the partial prevalence $\rho _{k}(t)$ ($\rho _{b}(t)$, $\rho _{h}(t)$ and $\rho _{s}(t)$) as the fraction of infected nodes with a given degree *k* degree(betweenness *b*, H-index *h*, and coreness *s*). The goal is to understand the propagation process under the measurement of four different node importance metrics on the epidemic processes. We compare the prevalence of the four different propagation dynamics. In the heterogeneous network, let *P*(*k*) denote the fraction of infected nodes that a node with a given degree *k* and $P(k^{'}|k)$ be the conditional probability that a node of degree *k* is connected to a node of degree $k^{'}$. The normalization conditions $\sum _{k}P(k)=1$ and $\sum _{k}P(k^{'}|k)=1$ hold. The average number of links connecting a node of degree *k* to some nodes of degree $k^{'}$ is $kP(k^{'}|k)$. Thus, the dynamic evolution processes can be written as

1 $$\begin{aligned} \begin{aligned} \frac{d\rho _{k}(t)}{d t}=-\rho _{k}(t)+\lambda k(1-\rho _{k}(t))\Theta _{k}(t), \\ \end{aligned} \end{aligned}$$

where

2 $$\begin{aligned} \begin{aligned} \Theta _k(t)=\frac{1}{\langle k\rangle }\sum _{k^{'}}k^{'}P(k^{'})\rho _{k^{'}}(t). \end{aligned} \end{aligned}$$

The first term on the right-hand side denotes that infected nodes of degree *k* can be recovered. The second term indicates that susceptible nodes of degree *k* are infected by their infected neighbors, where $1-\rho _{k}(t)$ represents the fraction of susceptible nodes of degree *k*, and $\Theta _{k}(t)$ is the probability that a link emanating from the nodes of degree *k* points to an infected node in the complex network. $\langle k\rangle$ is the first moment of degree distributions. When the right-hand side of Eq. (1) is zero, we obtain

3 $$\begin{aligned} \begin{aligned} { \rho _{k}=\frac{k\lambda \Theta _{k}(t)}{1+k\lambda \Theta _{k}(t)}, }\\ \end{aligned} \end{aligned}$$

Based on Eqs. (1) and (2), then one obtains

4 $$\begin{aligned} \begin{aligned} {\Theta _{k}(t)=\frac{1}{\langle k\rangle }\sum _{k}kP(k)\frac{k\lambda \Theta _{k}(t)}{1+k\lambda \Theta _{k}(t)},} \\ \end{aligned} \end{aligned}$$

The equilibrium point of endemic diseases in Eq. (1) must meet the following conditions:

5 $$\begin{aligned} \begin{aligned} {\frac{d}{d\Theta _{k}(t)}=\bigg (\frac{1}{\langle k\rangle }\sum _{k}kP(k)\frac{k\lambda \Theta _{k}(t)}{1+k\lambda \Theta _{k}(t)}\bigg )\bigg |_{\Theta _{k}=0}\ge 1,} \\ \end{aligned} \end{aligned}$$

one gets

6 $$\begin{aligned} \begin{aligned} {\sum _{k}\frac{kP(k)\lambda k}{\langle k\rangle }=\frac{\langle k^2\rangle }{\langle k\rangle }\lambda \ge 1,} \\ \end{aligned} \end{aligned}$$

where $\langle k ^2 \rangle$ is the second moment of degree distributions.

The epidemic threshold of heterogeneous network based on degree is^16^

7 $$\begin{aligned} \begin{aligned} { \lambda _c^k=\frac{\langle k \rangle }{\langle k^2 \rangle },} \\ \end{aligned} \end{aligned}$$

For the metrics of H-index, coreness, and betweenness centrality, the dynamic processes are constructed as follows:

8 $$\begin{aligned} \begin{aligned} \frac{d\rho _{h}(t)}{d t}=-\rho _{h}(t)+\lambda h(1-\rho _{h}(t))\Theta _{h}(t), \\ \end{aligned} \end{aligned}$$

where $\Theta _h(t)=\frac{1}{\langle h\rangle }\sum _{h^{'}}h^{'}P(h^{'})\rho _{h^{'}}(t)$, *h* is the metric for the nodes of H-index *h*.

9 $$\begin{aligned} \begin{aligned} \frac{d\rho _{s}(t)}{d t}=-\rho _{s}(t)+\lambda s(1-\rho _{s}(t))\Theta _{s}(t), \\ \end{aligned} \end{aligned}$$

where $\Theta _s(t)=\frac{1}{\langle s\rangle }\sum _{s^{'}}s^{'}P(s^{'})\rho _{s^{'}}(t)$, *s* is the metric for the nodes of coreness *s*.

10 $$\begin{aligned} \begin{aligned} \frac{d\rho _{b}(t)}{d t}=-\rho _{b}(t)+\lambda s(1-\rho _{b}(t))\Theta _{b}(t), \\ \end{aligned} \end{aligned}$$

where $\Theta _b(t)=\frac{1}{\langle s\rangle }\sum _{b^{'}}b^{'}P(b^{'})\rho _{b^{'}}(t)$, *b* represents the number of shortest paths through node *i*. Node betweenness is defined as the ratio of the number of shortest paths through node *i* to the total number of shortest paths in the network. We use the number of shortest paths through node *i* as metrics for modeling.

For the metrics of H-index, coreness, and betweenness centrality, by the same methods, we can conclude that the models have the following epidemic thresholds:

11 $$\begin{aligned} \begin{aligned} \lambda _c^h=\frac{\langle h \rangle }{\langle h^2 \rangle }, \\ \end{aligned} \end{aligned}$$

where $\langle h\rangle$ is the first moment and $\langle h ^2 \rangle$ is the second moment of H-index distributions.

12 $$\begin{aligned} \begin{aligned} \lambda _c^s=\frac{\langle s \rangle }{\langle s^2 \rangle }, \\ \end{aligned} \end{aligned}$$

where $\langle s\rangle$ is the first moment and $\langle s^2 \rangle$ is the second moment of coreness distributions.

13 $$\begin{aligned} \begin{aligned} \lambda _c^b=\frac{\langle b \rangle }{\langle b^2 \rangle }, \\ \end{aligned} \end{aligned}$$

where $\langle b\rangle$ is the first moment and $\langle b^2 \rangle$ is the second moment of betweenness distributions.

### Optimized immunization strategies

For epidemic spreading in complex networks, the immunization procedure can effectively prevent the spread of disease. Given that the proportional immunization schemes can effectively increase the immunity threshold, the special network of scale-free networks allows for efficient strategies on the basis of the hierarchy of nodes. Scholars have designed targeted immunization schemes where the highly connected nodes (i.e., nodes are likely to spread disease) are gradually immunized. Choosing the most effective metric to identify the most efficient spreaders for targeted immunization in a network is an important step. In contrast to common belief, the best spreaders correspond to the most highly connected or the most central people. In this study, four metrics (betweenness, degree, H-index, and coreness) are used to identify the most efficient spreaders for targeted immunization. For a fixed infection rate $\lambda$, the control parameter is the immunity proportion *g*, which is defined as part of the immune nodes that exist in the network. For the mean-field model, the presence of immunity can reduce the infection rate $\lambda$ by the factor $(1-g)$. By substituting $\lambda \rightarrow \lambda (1-g)$ in Eq. (3), the prevalence behavior of immunization rates is increasing. Consider that a proportion *g* of the nodes with the highest connectivity are immunized. This process corresponds to introducing an upper threshold $k_t$, such that all nodes with connectivity $k>k_t$ are immunized. The proportion of immunized nodes is given by Eq. (3) at the mean-field level, and the presence of immunity will reduce the infection rate $\lambda$ by the factor.

14 $$\begin{aligned} \begin{aligned} g=\sum _{k>k_t}P(k). \\ \end{aligned} \end{aligned}$$

For the metrics of H-index, coreness, and betweenness, the fraction g of the immunized nodes is given as follows:

15 $$\begin{aligned} \begin{aligned} g=\sum _{h>h_t}P(h), g=\sum _{s>s_t}P(s), g=\sum _{b>b_t}P(b). \end{aligned} \end{aligned}$$

### Data description

Four real networks and two BA scale-free networks from different fields are used to simulate the performance of the four models in Eqs. (3), (8), (9) and (10), including two communication networks (Email, ego-Facebook), one social networks (Political blogs), one transportation network (USAir), and two BA scale-free networks. Email^36,37^ describes the email interchanges between various users. Political blogs^38,39^ is a network between weblogs on US politics. ego-Facebook^40,41^ is collected from survey participants using the Facebook app. USAir^42^ is a network that describes US air transportation. Two scale-free networks are formed using the algorithm proposed by Barabási and Albert^25^ with different parameters. The topological features of these networks, including the number of nodes and links, average degree, average distance, and assortative coefficient, are shown in Table 1.

**Table 1:** The topological features of the six networks.

Networks	N	E	$\langle k\rangle$	$\langle d\rangle$	r
Email	1005	4855	9.622	3.61	0.078
Polblogs	1222	16,714	27.35	2.740	− 0.200
USAir	332	2126	12.81	2.738	− 0.208
Ego-facebook	4039	88,234	43.6	3.670	− 0.182
Scale-free network 1	5,000	150,000	60	2.670	− 0.01
Scale-free network 2	10,000	1,000,000	200	3.24	− 0.011

The features of these networks, including the max betweenness, max degree, max H-index, and max coreness, are shown in Table 2. For each network, the max betweenness of nodes is the largest, followed by max degree, max H-index, and max coreness. Therefore, the node importance fluctuations based on betweenness are the largest, followed by those fluctuations based on degree, H-index, and coreness. This finding implies that the most fluctuant metric is betweenness, followed by degree, H-index, and coreness.

**Table 2:** The maximum value of the four metrics.

Networks	Betweenness	Degree	H-indx	Core
Email	44,013	140	75	34
Polblogs	92,228	143	73	36
USAir	11,376	58	32	23
Ego-facebook	778,993	223	148	97
Scale-free network 1	82,724	343	131	78
Scale-free network 2	203,277	741	270	152

## Results

### Numerical simulations for epidemic model

**Figure 1:**
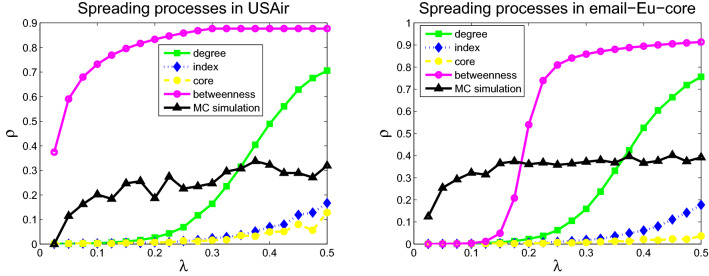
Infections prevalence $\rho$ as a function of spreading rate $\lambda$ for USAir (left) and email-Eu-core (right).

**Figure 2:**
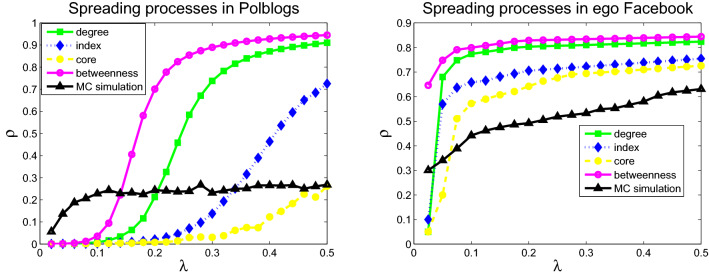
Infections prevalence $\rho$ as a function of spreading rate $\lambda$ for Polblogs (left) and ego-Facebook (right).

**Figure 3:**
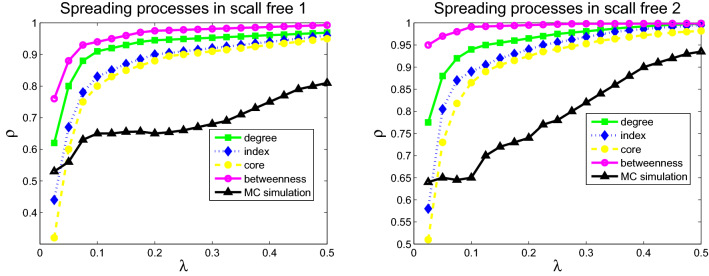
Infections prevalence $\rho$ as a function of spreading rate $\lambda$ for scale-free 1 (left) and scale-free 2 (right).

To study the present mean-field methods based on the four metrics, the numerical simulations of the SIS model on the six networks are used to display the spread dynamic process. The infection prevalence in the stationary state as a function of spreading rate $\lambda$ was simulated. The initial fraction of infected nodes is set to 0.05 (without lack of generality, we set the fixed probability of curing $\mu =1$). The comparisons of infection prevalence $\rho$ of the infected nodes among the four models and Monte Carlo simulations for six networks are displayed in Figs. 1, 2 and 3. Different disease spreading models exhibit diverse spreading processes. The model based on betweenness centrality exhibits the highest infection prevalence for all six networks, followed by the model based on degree, H-index, and coreness. Monte Carlo simulations results (black solid curve and triangle) were displayed in Figs. 1, 2 and 3. In the two networks (USAir and Email) with small number of nodes and small average degree, the variance between Monte Carlo simulation results and simulation results based on degree distribution is the smallest. In the other four networks (polblogs, ego-Facebook, scale-free network 1 and scale-free network 2) with large number of nodes and large average degree, the variance between Monte Carlo simulation results and simulation results based on coreness distribution is the smallest. Note that in the networks (ego-Facebook, scale-free network 1 and scale-free network 2) with large number of nodes, infections prevalence of Monte Carlo simulation is smallest compared with the four other models.

### Comparing the epidemic thresholds of epidemic models

Since more fluctuant metric lead to larger second moment, one can obtain:

16 $$\begin{aligned} \begin{aligned} {\langle b^2 \rangle>\langle k^2 \rangle>\langle h^2 \rangle >\langle s^2 \rangle }, \end{aligned} \end{aligned}$$

which imply that the model based on betweenness centrality exhibits the smallest epidemic threshold for all six networks, followed by the model based on degree, H-index, and coreness. Therefore, we can conclude that:

17 $$\begin{aligned} \begin{aligned} \lambda _c^b<\lambda _c^k<\lambda _c^h<\lambda _c^s. \end{aligned} \end{aligned}$$

**Figure 4:**
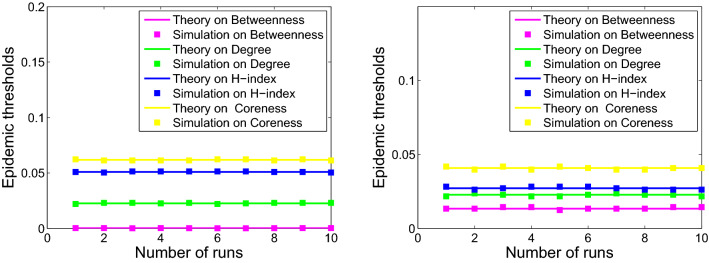
Numerical simulation (squares) and theoretical (solid curve) results of epidemic thresholds for USAir (left) and email-Eu-core (right).

**Figure 5:**
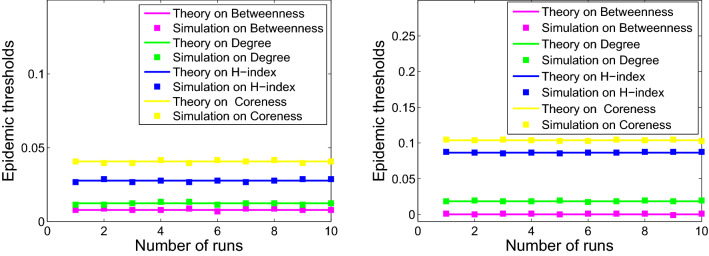
Numerical simulation (squares) and theoretical (solid curve) results of epidemic thresholds for Polblogs (left) and ego-Facebook (right).

**Figure 6:**
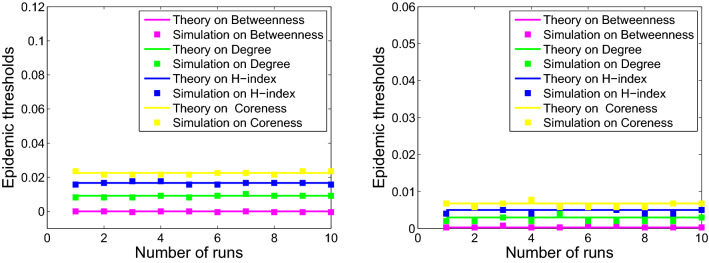
Numerical simulation (squares) and theoretical (solid curve) results of epidemic thresholds for scale-free 1 (left) and scale-free 2 (right).

The comparison of the epidemic thresholds for the networks from theoretical analysis described in Eqs. (7), (11), (12), and (13) with that from numerical simulations are displayed in the Figs. 4, 5 and 6. It shows that the theoretical analysis agrees well with numerical simulations with some minor deviation. Disease spreading models based on different metrics exhibit diverse spreading processes. Moreover, the simulation results are consistent with that of the previous analysis in Eq. (17).

### Comparing immunization effect of different immunization strategies

**Figure 7:**
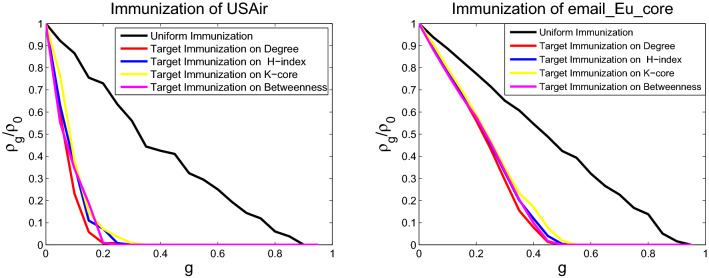
Comparison of the prevalence $\rho _g/\rho _0$ as a function of immunity proportion *g* with uniform and four targeted immunization for USAir (left) and email-Eu-core (right), at a fixed spreading rate $\lambda$ = 0.15.

**Figure 8:**
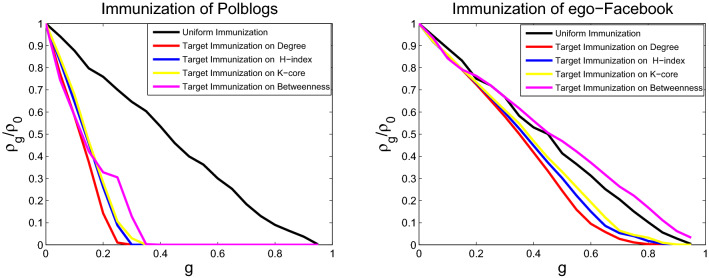
Comparison of the prevalence $\rho _g/\rho _0$ as a function of immunity proportion *g* with uniform and four targeted immunization for Polblogs (left) and ego-Facebook (right), at a fixed spreading rate $\lambda$ = 0.15.

**Figure 9:**
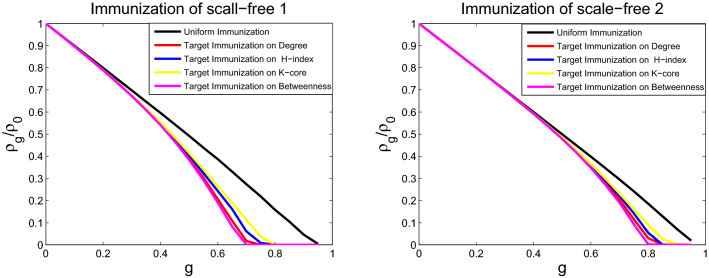
Comparison of the prevalence $\rho _g/\rho _0$ as a function of immunity proportion *g* with uniform and four targeted immunization for scale-free 1 (left) and scale-free 2 (right), at a fixed spreading rate $\lambda$ = 0.15.

The immunization behaviors for the SIS model on six networks are studied through numerical simulations for different immunization schemes. The targeted immunization scheme and uniform immunization are tested by immunizing the *gN* nodes. For a network of fixed size *N*, the uniform immunization is conducted by randomly selecting and immunizing *gN* nodes, and the targeted immunizations are performed by choosing the most central *gN* nodes to immunize. Node centrality is identified from betweenness, degree, H-index, and coreness centralities. The nodes at the top of the lists will be maintained. Infection prevalence $\rho _g$ in the stationary state as a function of different immunization proportion *g* is obtained through numerical simulation. The initial fraction of infected nodes is set to 0.05, the fixed spreading rate is $\lambda =0.15$, and the fixed probability of curing is $\mu =1$. The infection prevalence is computed by averaging over 100 runs of each model. Figures 7, 8 and 9 display the behavior of infection prevalence $\rho _g/\rho _0$ (where $\rho _0$ is the prevalence without immunization) as a function of immunization proportion *g*. For uniform immunization, the prevalence experiences a passive drop and exhibits the onset of large immunization threshold. On the contrary, the infection prevalence experiences an extremely sharp drop and exhibits the onset of small immunization threshold for targeted immunization (immunization threshold denotes that $\rho _g/\rho _0$ is 0 in Figs. 7, 8 and 9). The targeted immunization strategy using betweenness centrality to identify the most important nodes displays different immune effect for real networks, it displays the second best effect for USAir and email-Eu-core networks, as shown in Fig. 7, and displays the worst target immune effect among the four strategies, as shown in Fig. 8 for Political blogs and ego-Facebook networks. But it displays the best target immune effect for the two BA scale-free networks in Fig. 9. Excluding the target immunity based on betweenness centrality. For all networks, the target immunity strategy using degree centrality shows the most effective immune effect and obtains the smallest immunization threshold, followed by the model using H-index centrality, and the third is the model using coreness centrality.

## Discussion

**Figure 10:**
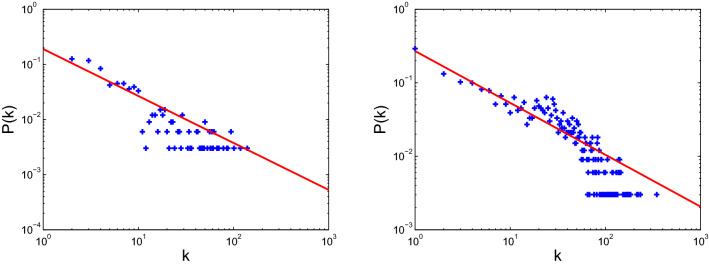
Degree distribution of USAir (left) and email-Eu-core (right).

**Figure 11:**
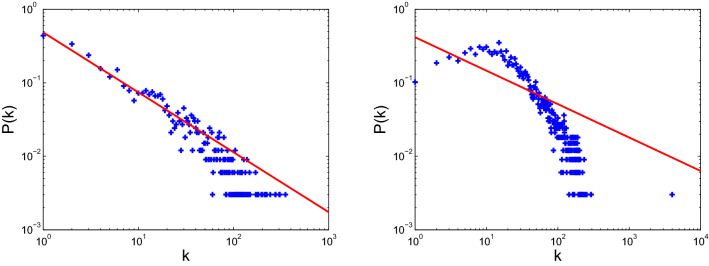
Degree distribution of Polblogs (left) and ego-Facebook (right).

**Figure 12:**
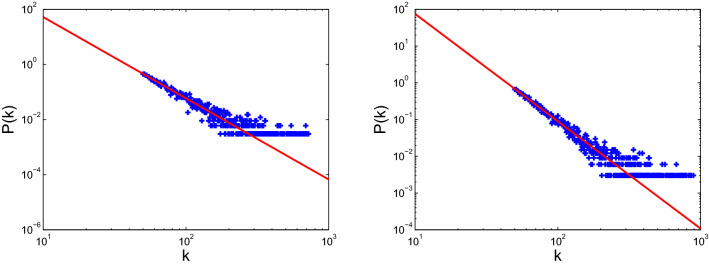
Degree distribution of scale-free 1 (left) and scale-free 2 (right).

The models for disease propagation based on betweenness exhibit the best propagation effect, followed by those models using the metrics of degree, H-index, and coreness. The models for targeted immunization based on betweenness exhibit the best immunization effect on BA scale-free networks, followed by those models using degree, H-index, and coreness. The targeted immunizations based on betweenness exhibit inconsistent effect for real networks because the node degrees of these real networks do not conform to the standard power-law distribution. The node degree distributions of these real networks and two BA scale-free networks are displayed in Figs. 10, 11 and 12. The right panels of Figs. 8 and 11 display that ego-Facebook whose node degrees are the least consistent with the power-law distribution shows the worst target immunity effect, and Figs. 9 and 12 show that the two BA scale-free networks whose node degrees are the most consistent with the power-law distribution show the best target immunity effect. The target immunization based on the node betweenness exhibits the best immunization effect on the network with standard power-law distribution (that is, the node degree distribution is a straight line in the double logarithmic scales). The models for disease propagation and targeted immunization based on degree exhibit the best propagation and immunization effects, followed by those models using H-index and coreness when the optimal of point betweenness is excluded. The key to obtaining these conclusions is the heterogeneity of node centrality. The greater the centrality disturbance of the nodes is, the more favorable it is to propagate and find the target immune nodes. The node importance fluctuations based on betweenness are the largest, followed by those fluctuations based on degree, H-index, and coreness. The results of this study are consistent with the conclusions in paper^26^, where degree, H-index, and coreness are the initial, intermediate, and steady states of the sequences operated by operator ${\mathscr {H}}$, respectively. Operator ${\mathscr {H}}$ makes strong fluctuations in the degree distribution to moderate fluctuations in the H-index distribution and abstains weak fluctuations in the coreness distribution. The simulations confirm that the most fluctuant metric is effective in disease propagation and identifying the spreading influences of nodes for target immunization. These results reveal that the largest fluctuant metric can be used to construct the fastest disease spreading model and identify the influential spreaders for target immunization. For networks with standard power law distribution, the best immune effect can be obtained by finding the most influential spreaders using the betweenness centrality as the immune target. However, for many real networks with nonstandard power law distribution, the best immune effect can be obtained using the degree centrality to find the most influential spreaders as the immune target. The 2019 Coronavirus Disease (COVID-19) has caused an outbreak on a global scale, so it is necessary to investigate control strategies to develop health care plans. The findings of this paper can guide people to accurately find the targets that need immunization, so as to effectively control the spread of the epidemic.

## Conclusion

The models for disease propagation and target immunization strategies based on node betweenness, degree, H-index, and coreness metrics are proposed in this study. The models for disease propagation based on betweenness always exhibit the best propagation effect, followed by those using the metrics of degree, H-index, and coreness. The models for targeted immunization based on betweenness exhibit the best immunization effect in BA scale-free networks, followed by those using degree, H-index, and coreness. However, the models for targeted immunization in four real networks based on node degrees exhibit the best immunization effect, while that based on betweenness for different real networks exhibit inconsistent effects (second-best or worst effects) because the node degrees of these real networks do not conform to the standard power-law distribution. The results provide reference for the public health sector to put forward effective measures for disease prevention and control.

## Data Availability

All relevant data can be downloaded from their respective web pages.
